# Toward resourcefulness: pathways for community positive health

**DOI:** 10.1177/17579759211051370

**Published:** 2021-11-22

**Authors:** Laura E. R. Peters, Geordan Shannon, Ilan Kelman, Eija Meriläinen

**Affiliations:** 1 Institute for Risk and Disaster Reduction, University College London, London, UK; 2 Institute for Global Health, University College London, London, UK; 3 College of Earth, Ocean, and Atmospheric Sciences, Oregon State University, Corvallis, OR, USA; 4 Stema Health Systems, London, UK; 5 University of Agder, Kristiansand, Norway

**Keywords:** assets/protective factors, capacity building (including competencies), communities, empowerment/power, equity/social justice, health promotion, policy/politics, salutogenesis

## Abstract

Communities are powerful and necessary agents for defining and pursuing their health, but outside organizations often adopt community health promotion approaches that are patronizing and top-down. Conversely, bottom-up approaches that build on and mobilize community health assets are often critiqued for tasking the most vulnerable and marginalized communities to use their own limited resources without real opportunities for change. Taking into consideration these community health promotion shortcomings, this article asks how communities may be most effectively and appropriately supported in pursuing their health. This article reviews how community health is understood, moving from negative to positive conceptualizations; how it is determined, moving from a risk-factor orientation to social determination; and how it is promoted, moving from top-down to bottom-up approaches. Building on these understandings, we offer the concept of ‘resourcefulness’ as an approach to strengthen positive health for communities, and we discuss how it engages with three interrelated tensions in community health promotion: resources and sustainability, interdependence and autonomy, and community diversity and inclusion. We make practical suggestions for outside organizations to apply resourcefulness as a process-based, place-based, and relational approach to community health promotion, arguing that resourcefulness can forge new pathways to sustainable and self-sustaining community positive health.

## Introduction

The role of communities in promoting health and wellbeing is a tenet of public health research and practice. While recognizing that communities are not homogenous or static (1), communities can be linked by common interests and conditions, becoming effective and successful agents of change regarding the connected and complex global and local challenges affecting their health. This article asks to what extent outside organizations can support communities to forge pathways to positive health, and explores community health promotion approaches that reinforce community agency and self-determination and ultimately contribute to reducing global health disparities and inequities. We use the terms ‘agency’ and ‘agency-based approaches’ to refer to community agency and not in reference to organizations or institutions. We provide a critical examination of how community health may be defined and determined, as well as an overview of community health promotion including with respect to the advantages and critiques of agency-based approaches. We offer and explore the concept of ‘resourcefulness’ to provide insights into how positive health can be approached as a relational and place-based process and discuss the role(s) that outside organizations can play in supporting communities to strengthen their health assets, renegotiate power relationships, and cultivate local human–environment relationships that form the basis for future health choices, opportunities, and potentialities.

## Conceptualizing community positive health

How health is conceptualized instructs where, when, and how it is promoted. Yet, the concept of health is under-scrutinized and often used uncritically in the field of public health (2). The biomedical illness model has dominated contemporary health practices and approaches and shaped Western understanding and practice of health as ‘normal functioning’ and the absence of disease (3). However, health can be understood as a presence of positive features rather than merely an absence of negative ones. This concept underpins the World Health Organization’s (4) definition of health as ‘[a] state of complete physical, mental and social well-being and not merely the absence of disease or infirmity’. This definition lends itself to ‘positive health’, which we conceptualize as a spectrum of wellbeing and flourishing partially independent from disease or infirmity that is determined by a collection of health assets. The effects of positive health can be seen in increased longevity and improved abilities to recover from health challenges (5), as well as in broader perceptions of social, cultural, and emotional wellbeing. An expanded notion of health illuminates that it is normatively, rather than objectively defined (6,7).

Several theories of health provide for a deeper understanding of positive health. Nordenfelt (8) proposed health as the ability to achieve vital goals that secure a person’s minimal happiness in the long run and achievement of a life that is minimally decent. From this perspective, happiness is relative to a person’s set of goals and their ability to achieve them, which is derived from their agency, circumstances, and environment (6,8). Building on the work of Sen (9,10) and Nussbaum (11–13), a capabilities approach to health is ‘conceptualized as her abilities to be and do things that make up a minimally good, flourishing and non-humiliating life (7)’. Ability and capability theories both emphasize the non-standardized health goals that people manage to achieve as well as meaningful opportunities to pursue their health.

These ideas complement those from diverse cultures and places. For example, various Indigenous conceptualizations of health emphasize culture, spirituality, interdependence, and interconnections between the individual and their wider environment. In Australia, Aboriginal and Torres Strait Islander social and emotional wellbeing sits across mental, physical, family, community, cultural, spiritual, and environmental and place-based health factors (14). In First Nations communities in Canada, positive health, or ‘thriving’, is associated with interdependence and interconnectedness across social, family, and community support (15). The Andean concept of Sumaq Kawsay, or Buen Vivir, outlines a plurality of understandings that emphasizes collective wellbeing and living in harmony with others and the natural environment (16). These approaches to health that predate mainstream global health are often marginalized by contemporary practice, but they make important contributions to understanding positive health for communities, or ‘community positive health’.

These approaches not only resist reductionist and individualistic (i.e. biomedical) notions of health, but they also articulate the value of collective community health outcomes. While mainstream global health practice tends to treat community health as individual-level health data in aggregate, collective notions of wellbeing are greater than the sum of individual health achievements alone and even call into question the strict delineation between individual, interpersonal, and family health in more collectivist societies (17). Communities are positioned as central to positive health, and community health holds important value instrumentally (as a means to promote individual health and facilitate salutogenic processes) and inherently (as a collective value or public good).

Health is commonly seen as being determined by interactions between internal biology, human behavior, the external physical environment, and social conditions (18,19). The ecological model highlights that health influences occur at multiple interacting scales, including public policy, community, institutional, interpersonal, and intrapersonal levels (20). In this framing, communities occupy a vital bridging position between larger and smaller scales of health. On the one hand, the community shapes individual and family level health outcomes: collective conditions – including resource constraints and symbolic power (21) – influence access to health and shape the immediate physical and non-physical environment. On the other hand, the community sits at the interface between individuals and broader structural influences: communities may buffer or augment socioeconomic, geopolitical, or environmental determinants of health.

Studying the multiple causes of health, or salutogenesis (22), may reveal distinctions from the multiple causes of diseases and other health challenges and deepen an exploration of not only how to prevent or recover from disease or illness but also how to strengthen the building blocks and patterns of positive health. Although the *determinants* of health enable some understanding of complex salutogenic systems, critical epidemiologists such as Spiegel *et al*. (23) call for a shift away from ‘risk factor’ dialogue to one that examines process and power, drawing from critical Latin American scholarship to make an argument for the ‘social determination’ of health. This signals a paradigm shift in how health is conceptualized and achieved: individuals and groups move away from being passive entities that experience discrete health risk factors toward being agents in creating their health. This agency reflects the ability of communities to navigate dynamic and complex systemic health influences and respond to broader structural challenges in order to create pathways to positive health.

## Pathways to community positive health

Health promotion seeks to increase access to health by reducing health inequities and inequalities and increasing health opportunities (24), and a focus on what sustains health is reflected in global policy, such as the Ottawa Charter (25), which lays the groundwork for people to take control of their own health (26). Community health promotion ideally strives to build community agency and self-determination, where communities define their own health values and goals and determine how to work toward them rather than passively receiving public health decisions and interventions. Even the most vulnerable and marginalized communities should be characterized by more than their needs, challenges, and limitations, and recognized for the central role they play in strengthening their own health.

However, in practice, community health promotion approaches have tended to focus on addressing narrowly defined needs and improving specific health outcomes through top-down solutions (27). These technology-centered and/or disease-specific ‘fixes’ have been deemed ineffective and unsustainable (27,28), as they overlook the multiple dimensions that undergird long-term health and wellbeing. Further, they do not factor in broader conceptualizations of positive health or engage with communities as partners and leaders in developing and implementing health strategies.

Faced with the shortcomings of conventional community health promotion in practice, agency-based approaches derived from community develop-ment research and work emphasize building community strengths for health rather than focusing solely on needs and deficits. Asset-based approaches focus on what a community believes is important for their health (29) and include physical as well as intangible resources related to individual, social, environmental, and political factors (30) and their interactions (31). Asset-based approaches aim to help communities identify their key resources, build and nurture them, connect and reinforce them, and/or leverage them to achieve self-identified health goals (32,33). Because health resources emerge from within communities, asset-based approaches do not call for heavy-handed external input, including the introduction of new technologies.

While asset-based approaches center on the *objects* of health, community mobilization (CM) further recognizes and supports the power within rather than beyond the community by emphasizing *process*. CM is ‘a capacity-building process through which community individuals, groups, or organizations plan, carry out, and evaluate activities on a participa-tory and sustained basis to improve their health and other needs, either on their own initiative or stimulated by others (34)’, which has been practiced in connection with emancipatory community development work occurring across Latin America (35,36). CM focuses on cultivating *participation* (community and other stakeholders), *partnerships* (with supportive outside actors), and *powe*r (37,38) to stimulate needed changes. Much practice has been informed by Freire (39) and involves a dialogical process of change, where a reflection–action cycle promotes critical thinking and ‘empowerment’ of marginalized com-munities (37,38).

These agency and empowerment-based approaches to health related to *object* or *process* have the potential to liberate communities from cycles of aid chiefly through empowering communities to reimagine and reinvent themselves out of a passive or victim role in which they may have been placed. These approaches have nonetheless been critiqued for focusing squarely on developing the innate characteristics of communities while broadly failing to address the structural causes of health that systemically perpetuate inequities (26,40), and they seek to build a healthier future without recognizing the histories and patterns that determine current conditions. The burden of change falls on the most marginalized and disadvantaged communities, and they may face blame for failing to ‘choose’ to be healthy (41,42) even when social, political, economic, and/or environmental impedi-ments make it virtually impossible to achieve targeted health objectives. Nordenfelt (6) argued that psychological routes to health may be the only viable option when political solutions to structural health challenges are not available, but this defense falls short of identifying how communities are meant to bypass structural and historical constraints to make meaningful health improvements and reduce health inequities.

## Community positive health through resourcefulness

Community health defined positively may be closely tied with how communities can foster and leverage their collective strengths both to overcome complex health challenges and pursue their health. Global processes and events – including environmental changes, structural racism and sexism, violent conflict, and vulnerabilities causing other disasters – combine with and are influenced by community-level factors, and altogether these shape dynamic challenges and opportunities for health. The reciprocal interplay between factors at multiple scales (43) underlines that community health promotion must target structural changes to achieve more equitable resource distribution (44) while also empowering communities to guide these structural changes in ways that bolster their agency.

The concept of resourcefulness offers useful and usable insights into pursuing and leveraging community positive health in constraining and dynamic contexts. In critiquing mainstream resilience discourse, MacKinnon and Derickson (45) developed the idea of resourcefulness as a relational and place-based process – rather than a condition or characteristic that communities may or may not possess to some degree – that is centrally interested in the generative nature of communities. Resourcefulness-based approaches aim to foster and mobilize material and non-material resources, skill sets and technical knowledge, Indigenous and folk knowledge, and recognition to enable positive changes based on community priorities and needs (45). Recognizing the uneven distribution of material resources and power that induces resource scarcity and systemically disadvantages certain groups and communities, resourcefulness stresses that the changes necessary for community flourishing are not socially or politically neutral.

Applied to community positive health, resource-fulness has the potential to forge new multipronged pathways for promoting sustainable and self-sustaining community positive health. Through resourcefulness-based approaches, communities cultivate the agency to (a) conceptualize what constitutes their health and health assets and (b) pursue and sustain health agendas driven by local priorities, needs, and learning, while they also work to (c) change power imbalances that drive inequitable patterns of material resource distribution, and (d) nurture ecologically sound relationships with their local environment. Communities pursue their health through their internal strengths and supplement them through strategic partnerships: partners may include other communities with similar priorities and/or complementary resources and other actors at higher institutional scales with additional resources and power. By creating ‘genuinely deliberative democratic dialogue’ and developing ‘contestable alternative agendas’ (45), communities are able to take an active and intentional role in determining their health and challenge the systemic drivers of health inequities. Resourcefulness-based approaches thus combine agency-based approaches focused on objects and processes and interweave them with structural approaches to health promotion. In keeping futures and potentialities as well as histories and patterns in full view, resourcefulness recognizes both the continuity and dynamism of health.

Resourcefulness-based approaches offer insights into three interrelated aspects within community health promotion:

1) Resources and sustainability: a community’s resourcefulness has the potential to offset certain material resource deficits (46), but natural and other material resources are understood as necessary inputs not only for health but also to access the levers of change. Economic growth and development in aggregate is not the answer to ending (and may indeed promote) disease and illness and their drivers of poverty, marginalization, and inequities, as those with the most power tend to capture the majority of the benefits and evade the costs. Where transforming natural resources into goods and services for health is necessary, it is done congruently with local human–environment relationships that form the basis for future health choices, opportunities, and potentialities, since extracted and degraded local resources that cannot be regenerated no longer function as health assets for communities. Resourcefulness thus recognizes the importance of environmental conservation and regeneration: when appropriate and possible, resourcefulness pursues less resource-intensive solutions to health challenges and opportunities; advocates for the equitable distribution of existing resources, goods, and services; and works with the natural environment.
2) Interdependence and autonomy: unlike purely agency-based approaches that focus on a community’s self-reliance and determination, the relational lens of resourcefulness recognizes the interconnectivity of communities with larger institutional and/or spatial scales (vertical linkages) as well as other communities (horizontal linkages). Strengthening progressive translocal connections may help to challenge inequitable relationships within broader systems of power, like dominant economic systems (45), and advance collaborative advocacy for collective rights and recognition from within and beyond the community. Working with partners with access to more resources and power can help to influence changes at higher scales and sustain local health agendas. Resourcefulness may, therefore, support self-help alongside targeted help-seeking from external sources to avoid the added injustice of responsibility without the power to act.
3) Community diversity and inclusion: communities are not homogenous and bounded entities, and community members do not share entirely unified values and goals (47). Members of a community may not be uniformly affected by local and global events and processes, and some may even stand to gain in the short term from drivers of inequity like extractive capitalism, further impeding collective action (48). Resourcefulness recognizes the central importance of broad community participa-tion and ownership in strategies for health and sees community heterogeneity – including diverse knowledge, perspectives, and skill sets – as a strength. Inclusive community positive health initiatives may foster new social connections, social innovation, and capacities for collective action (49) and, in doing so, contribute to more integrative communities and sub-communities of care and self-sustaining community positive health outcomes over the long term.

## Resourcefulness for community positive health in practice

The potential usefulness and usability of resourcefulness-based approaches for outside organiza-tions promoting community positive health can be found in diverse communities, settings, and issues, alongside their complications and shortcomings in practice. For example, resourcefulness may be leveraged to break dependencies that are harmful to the short- and long-term health of marginalized communities and forge more equitable power relations and interactions that are conducive to community positive health. Yet, systems are characteristically resistant to change, especially when some actors gain from inequitable resource distribution.

Community activism may be met with violence, which was the case with Honduran environmental activist and Indigenous leader Berta Cáceres. Cáceres organized local communities to peacefully resist the building of the internationally funded Agua Zarca Dam on the Gualcarque River, which is integral to the positive health of the Lenca People. Her coordination was recognized with a Goldman Environmental Prize in 2015, but her efforts also resulted in her assassination in 2016 (50). After this and other violent incidents received widespread international media coverage and galvanized collective demands for change, the funding and consequently the construction of the hydro project was eventually suspended. This is far from an isolated case, and Indigenous land and environmental defenders are killed or targeted with violence at disproportionately high rates (51). Large-scale hydropower development has experienced a resurgence in interest around the world, but the social and environmental impacts of these extractivist projects, including provoking social and environmental conflicts and deteriorating community health, are considerable (52). Local strategies for health may benefit from engaging with place while also connecting with transnational movements for advocacy and recognition (53), but equity is not merely freely available for those who choose to pursue it. This example highlights the barriers that prevent communities from pursuing their community positive health, and it also suggests potential supporting roles that outside organizations adopting resourcefulness may play, including providing outside legitimacy and financial resources to bolster ongoing community efforts, connecting communities with others in similar situations worldwide to share challenges and solutions, promoting international advocacy campaigns featuring community knowledge to foster broader awareness, and doing more to protect community leaders and activists.

Other situations highlight how outside organizations play a complicated hand in creating health challenges that play out in communities while also supporting community solutions. For example, the structural adjustment programs (SAPs) of the 1980s reduced public spending on health systems in economically marginalized countries, and international non-governmental organizations stepped in to patch the widening cracks (54). Meanwhile, the international aid community started channeling funding to combat the HIV/AIDS global epidemic (55). The combination of reduced government spending and a narrow non-governmental HIV/AIDS focus contributed to the neglect of primary healthcare and to siloed healthcare systems in places like Sierra Leone (54,56). The United Nations International Children’s Emergency Fund (UNICEF) and the World Bank launched the community health worker program in Sierra Leone to support primary healthcare (57), and community health workers received training and became responsible for various aspects of healthcare particularly in rural and otherwise marginalized communities (57,58). While the community health worker program is a problematic legacy of a donor-driven post-SAP era, it has expanded access and incorporated community concerns and priorities into the Sierra Leonean healthcare system. The practices remain diverse and connected with place-based concerns and priorities, despite their continued dependence on donor funding and national aims to increase regulation and uniformity (57,59).

The 2014–2016 Ebola outbreak in West Africa brought the important and enduring role of community health workers to the fore, as they were more effective than outsiders at Ebola response and were able to continue providing maternal and child health services alongside traditional birth attendants, community health committees, and traditional healers (59). This example shows that singularly focusing external resources on a narrow health problem can generate foundational challenges to community positive health. Communities are capable of identifying solutions for themselves. Expectations of regulation and uniformity from outside organizations may undermine the unique building blocks of positive health situated in specific communities. Resourcefulness offers lessons for outside organizations to support community positive health systems already in place rather than developing heavy-handed agendas outside of communities and delivering trainings without regard for existing skills and diverse forms of local knowledge.

These vignettes shed light on how communities demonstrate resourcefulness in developing necessary place-based community health strategies, but not without costs and complications. Communities can and do navigate a dynamic stream of challenges and creatively seize opportunities embedded within these challenges to strengthen their community positive health. At the same time, the combination of these multiscalar challenges most often leaves the most socially, politically, economically, and envi-ronmentally marginalized communities with increasingly fewer opportunities, resources, and capabilities to realize their health goals. Thus, these examples illustrate that while communities are powerful and necessary agents in determining and advancing their own health, they also benefit from partnerships and alliances – including with outside organizations – that collectively wield greater power and influence to create necessary changes. Resourcefulness as a process-based and relational practice depends on long-term relationships that adapt to the changing needs, goals, and conditions of communities as well as the dynamic challenges and opportunities they face.

Practical resourcefulness-based strategies for outside practitioners and policymakers to support community positive health may take many forms, including the following non-exhaustive list:

1) developing long-term relationships with com-munities that build trust over time through learning from and respecting community leaders and community mechanisms for problem-solving and planning;
2) supporting bonding within communities, con-necting with other communities, and linking with larger institutional scales to coordinate health strategies;
3) providing support in advocacy and lending perceptions of legitimacy to increase broader recognition of community health initiatives and strategies;
4) fostering awareness and knowledge about current and expected future environmental conditions and their impacts on natural resources;
5) providing seed funding or financial backing for experimentation, as well as continuing support for ongoing initiatives and the maintenance of relationships between communities and organi-zations over time;
6) assisting with problem-solving when health initiatives encounter barriers and challenges; and
7) providing a platform for inclusive internal and translocal knowledge creation and sharing.

The overarching purpose of these distilled resourcefulness-based strategies is to offer inroads for outside organizations to help support and strengthen community positive health in ways that complement and do not supplant existing community strengths, knowledge, and initiatives. Yet, recogni-zing that health challenges and constraints can stem from or intertwine with community-level factors, resourcefulness-based strategies may also include bringing to light new information, ideas, and other opportunities and resources to help forge new pathways to community positive health.

Outside organizations adopting a resourcefulness mindset may be better able to link their efforts and investments to health processes and changes that are locally meaningful and self-sustaining. Despite these benefits, outside community health promotion organizations and donors may be reluctant to embrace these roles, because they entail that communities retain or gain control of health promotion processes. Best practices that emerge in a specific context may not be appropriate to import into another context, and this constant learning process may challenge an organization’s ability to solidify evidence-based approaches and streamline projects. Community goals may not align neatly with the global public health agenda, progress toward them may not be linear or take place over the short term, and achieving them may not be easily captured through standard monitoring and evaluation tools.

Nevertheless, resourcefulness may be a viable way forward in light of health disasters like the COVID-19 pandemic. Resourcefulness-based strategies could arguably help with pandemic prevention and mitigation in the future by supporting the objects and processes of community positive health directly. When global prevention fails, local resourcefulness could be leveraged to keep integrated formal–informal health systems running, prevent health workers and others from dying from the disease and other treatable ailments, and mitigate its effects on other essential aspects of community positive health (e.g. food security, social cohesion, and information sharing). It might also then galvanize global and multiscalar resourcefulness for positive health.

## Conclusion

This paper has applied and adapted the concept of resourcefulness as a process-based, place-based, and relational approach to understand and pursue community positive health, with the goal of strengthening health opportunities and choices. Resourcefulness builds on bottom-up approaches by strengthening and mobilizing community assets, but it also seeks to address the structural factors that determine health by forging tools and partnerships through long-term cooperative actions at multiple scales. Recognizing the importance of material factors and natural resources in community positive health, resourcefulness-based approaches also emphasize the cultivation of socially and environmentally sustainable practices and relationships, and they challenge the inequitable power relations and environmental practices that degrade local resources and capacities for health. Future research may explore how resourcefulness-based approaches to community health promotion can be leveraged in applied settings to make meaningful gains in narrowing health equity gaps.
